# Treatment of Capitellum and Trochlear Fracture using Headless Screw with Concomitant Olecranon Fracture Through Posterior Approach: A Case Report

**DOI:** 10.5704/MOJ.2503.015

**Published:** 2025-03

**Authors:** NZA Aishah, HA Noor

**Affiliations:** Department of Orthopaedics, Hospital Sultanah Nur Zahirah, Kuala Terengganu, Malaysia

**Keywords:** trochlear, surgical approach, headless screw, distal humerus, coronal shear fracture

## Abstract

Articular fracture of distal humerus account for 2% of all adult elbow fracture. The coronal shear fracture combined with olecranon fracture require stable anatomical reduction and stabilisation for early rehabilitation and mobility. Numerous reports have described various approaches in tackling this type of fracture and method of fracture fixation. Here, we shared a case of a lady with traumatic coronal shear capitulum and trochlear comminuted fracture fixed using posterior elbow approach with fracture site open window technique in managing distal humerus articular fracture with ipsilateral olecranon fracture using headless screw and tension band wire. During last follow-up, fracture was united radiographically with congruent articular joint, patient able to return to her original function without limitation and no indications of avascular necrosis.

## Introduction

Distal humeral fracture denotes less than five percent of all adult elbow fractures^1^. McKee *et al*^2^ introduced the concept of coronal shear fracture that involves anterior and proximal displacement of capitellum, lateral ridge of trochlear and part of trochlear which is similar to our case. As it rarely occurs, producing a specific management outline is difficult. The main aim was to achieve maximum articular congruency with solid fixation.

Here, we report a case of olecranon and distal humerus coronal shear fracture treated by open reduction and headless screw for the distal humerus fracture and tension band wire for the olecranon fracture using posterior approach our way.

## Case Report

A 57-year-old right hand dominant housewife sustained closed comminuted fracture left distal humerus and olecranon following fall with direct force on her elbow at home. Post trauma, she sustained pain, swelling and limited range of motion of the left elbow, otherwise no injury elsewhere. Clinically the left elbow appeared swollen with a bruise over lateral elbow, the compartment remains soft with intact distal pulses and neurology, kept in flexion.

**Fig. 1:**
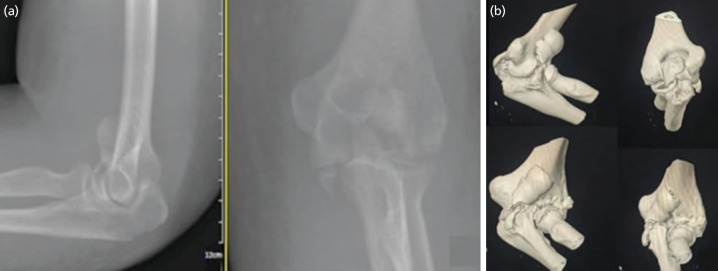
(a) AP and lateral radiograph of left elbow shows comminuted fracture of distal humerus. (b) Computed tomography of left elbow shows the trochlear fragment displaced anteriorly with ipsilateral olecranon fracture.

Radiograph film of left elbow shows comminuted fracture of left distal humerus and olecranon (Fig. 1). Computed tomography then further revealed comminuted fracture of humeral trochlear which displaced superior and anteriorly, capitulum and simple oblique fracture of olecranon and coronoid process without elbow dislocation (Fig. 1). She was put on a backslab and armsling for immobilisation and started on heterotopic ossification prophylaxis.

**Fig. 2:**
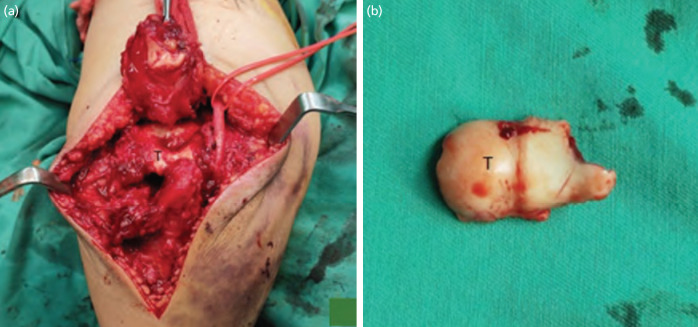
(a) Intra-operative image of fracture site with trochlear fragment reduced in place, (b) picture of trochlear fragment.

**Fig. 3:**
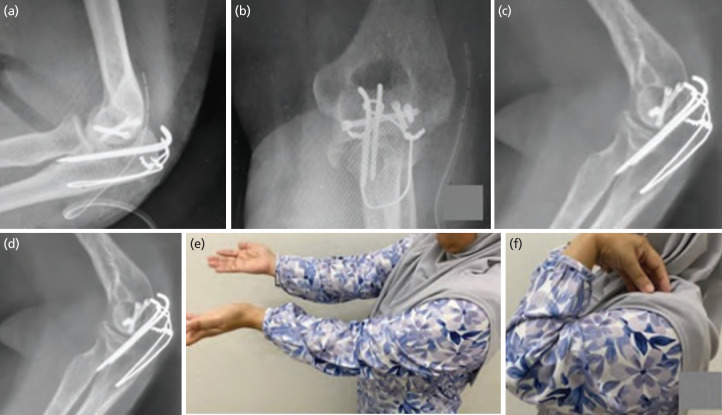
(a, b) Immediate post operative radiograph shows acceptable fracture fragment reduction with congruent articular surface. (c, d) United olecranon and coronal shear fracture with congruent articular surface at six months after fixation. (e, f) Post-operative range of motion.

Surgery was performed fourteen days following the initial injury. The patient was put in a lateral decubitus position with the upper arm supported by padded post. Longitudinal midline incision done over posterior elbow avoiding tip of olecranon to prevent skin irritation and for best possible view at the same time addressing the olecranon fracture. The deep fascia incised, and ulnar nerve is explored and protected with vessel loop. Soft tissue attachment over either side of olecranon stripped to assess fracture site. The distal part of humerus exposed by reflecting the triceps and proximal part of olecranon, debridement done (Fig. 2). The trochlear fracture fragment was displaced anterosuperiorly and was unable to be delivered. Therefore, a small window was created between the superomedial border of distal humerus and ulnar nerve to deliver the fragment. It was reduced and temporary fixed with k-wire followed by two headless screws sized 2.4mm in posterior-anterior direction and a rafting screw. Then, the olecranon was fixed with tension band wire using two 1.8mm k-wire and 1.2mm cerclage wire. The reduction and fixations were confirmed stable under image intensifier guidance. Post-operation, radiograph image (Fig. 3) revealed stable elbow fixation, patient was started on passive movement exercise after two weeks. During the last appointment, at eight-month post-operation, the fracture united, active range of motion of the elbow was about 5° to 120° with no varus valgus laxity (Fig. 3). The patient underwent home therapy and was satisfied with her current condition.

## Discussion

Distal humeral coronal shear fracture account for two percent of all fractures at the elbow level^3^. The most common pattern of osteochondral fracture of distal part of humerus involves the capitellum^2^. It is caused by direct force providing shear and axial load to capitellum and trochlea, usually due to fall on outstretched hand^4,5^. The combination mechanism for coronal shear fracture of the distal humerus with olecranon fracture such in our case also described by Sun *et al* (2019) and Yoshida *et al* (2021), caused by an axial loading force applied to a flexed elbow propagating into the distal humerus from the olecranon is unusual^3,4^.

The primary objective of distal humerus fixation is a perfectly stable fracture as this will enable early rehabilitation needed to regain normal mobility^1^. Articular surface fracture shall be distinguished from the single or bicolumn fracture by lacking of metaphyseal fracture extension proximal to olecranon fossa^5^. Although the exact morphology of these fractures could not be ascertained from the pre-operative radiographs and was not present in our case, the double arc sign, characterised by a half-moon shaped, separated from capitellum on lateral radiograph is sensitive in diagnosing coronal shear fracture^3,5^. We now make use of computed tomography with three-dimensional reconstructions to improve our pre-operative analysis of fracture pattern and planning optimal fixation.

A posterior approach with fracture site open window technique and fixation with posterior-to-anterior interfragmentary screws instead of usual manner of anterior-to-posterior direction are suggested as a surgical intervention by Sun *et al* for case of coronal shear fracture of distal humerus associated with olecranon fracture^3^. This technique was very similar to our case operative management. Intra-operatively, we have difficulty in delivering and reducing the trochlear fragment, thus we created a small window between superomedial border of distal humerus and ulnar nerve to deliver the piece from anterior to posterior. This technique has not been reported as to our knowledge. Dubberly classification may help us to decide surgical intervention, with absent of posterior comminution, treatment with only headless compression screw is reliable, while with posterior comminution fixation can be effective with addition of dorsal locking plate. In this case, the patient is Dubberly 2A where the fracture involves the capitellum in continuity with more extensive extension into the trochlea with no posterior comminution, thus headless compression screw is the gold standard and was shown to give excellent result^4^. Its double threaded design allows compact fixation of fracture site with both end of the screws buried beneath the bone surface. The surgery was challenging as we need to reduce the comminuted displaced fracture fragments, however we managed to reduce and obtain solid fixation with two headless screws.

Despite the patient showing good functional outcome during last follow-up, the average duration of follow-up for this type of fracture was about one year up to two years in view of risk for avascular necrosis. In our case, the follow-up was only eight-months as patient defaulted. It is still important to monitor occurrence of avascular necrosis of the capitulum as it may require hardware removal and is a limitation to our study.

In conclusion, we treated a case of comminuted distal humerus articular and olecranon fracture, Dubberly 2A. Approaching from posterior whenever having ipsilateral olecranon fracture is a good method to consider in managing distal humerus articular fracture with alternative method to reduce the anterosuperior fragment from fracture site by creating a new medial window plane. Not only able to preserve the articular congruency but post-operatively also shows satisfactory elbow function and follow-up result.
